# A Study on the Effect of the Structural Parameters and Internal Mechanism of a Bilateral Gate-Controlled S/D Symmetric and Interchangeable Bidirectional Tunnel Field Effect Transistor

**DOI:** 10.1186/s11671-021-03561-8

**Published:** 2021-06-08

**Authors:** Xiaoshi Jin, Yicheng Wang, Kailu Ma, Meile Wu, Xi Liu, Jong-Ho Lee

**Affiliations:** 1 School of Information Science and Engineering, Shenyang University of Technology, Shenyang, 110870 China; 2 School of EECS Engineering and ISRC (Inter-University Semiconductor Research Center), Seoul National University, Shinlim-Dong, Kwanak-Gu, Seoul, 151-742 Korea

**Keywords:** Tunnel field effect transistor, CMOS, Bidirectional switch, Subthreshold swing, Nanoscale

## Abstract

A bilateral gate-controlled S/D symmetric and interchangeable bidirectional tunnel field effect transistor (B-TFET) is proposed in this paper, which shows the advantage of bidirectional switching characteristics and compatibility with CMOS integrated circuits compared to the conventional asymmetrical TFET. The effects of the structural parameters, e.g., the doping concentrations of the N^+^ region and P^+^ region, length of the N^+^ region and length of the intrinsic region, on the device performances, e.g., the transfer characteristics, *I*_on_–*I*_off_ ratio and subthreshold swing, and the internal mechanism are discussed and explained in detail.

## Introduction

Power consumption is one of the main problems of the integrated circuit industry. If a device works in the on state, its conduction current must reach a certain critical value; when the current reaches a critical value, the corresponding gate voltage is defined as the threshold voltage. When the device is in the off state, the corresponding gate voltage should be a different value from that in the critical on state, which is often called the off-state voltage. The concept of subthreshold swing (SS) is applicable to the device that operates between the off state and the critical on state, which is equal to the change in gate voltage when the current increases by an order of magnitude. When the device is well designed, the critical on-state current value, threshold voltage and off-state voltage of the device have been determined; then, a smaller SS corresponds to stronger current changes in the subthreshold area, a smaller static current of the device in the off state, and lower static power consumption of the device. The SS of metal oxide semiconductor field effect transistors (MOSFETs), which are the basic unit cells widely used in integrated circuits, is limited by the physical mechanism of the current generated while the device is working and cannot be lower than the limit value of 60 mV/dec. To breakthrough this limitation, a tunnel field effect transistor (PIN or NIP TFETs) based on silicon-based technology has been proposed in recent years. A conventional TFET is formed by adding a layer of low doping intrinsic semiconductors between *p*- and *n*-type semiconductor materials. Compared with MOSFET, the TFET has the advantages of high sensitivity and low static power consumption [1]. TFET is switched by modulating quantum tunneling through a barrier instead of modulating the thermionic emission over a barrier as in the traditional MOSFET. Thus, TFET is not limited by the thermal Maxwell–Boltzmann tail of carriers, which limits the SS of MOSFET to 60 mV/dec at room temperature [2] (exactly 63 mV/dec at 300 K). The concept was proposed by Chang et al. while working at IBM [3]. For the first time, Joerg Appenzeller and his colleagues at IBM demonstrated that the SS of TFET could be lower than 60 mV/dec. TFET can be used as energy-efficient electronic switches [4], which breaks through the bottleneck of MOSFETs and greatly reduces the IC power consumption. The production process is compatible with MOSFETs. It is likely to replace the MOSFET transistor as the basic unit of next-generation integrated circuits. Therefore, TFETs have become a hot topic in recent years [5, 6]. To improve the performance of TFETs in terms of SS, forward conducting current and reverse leakage, many studies on the structure design and optimization of TFET devices have been conducted, which mainly focus on improving the structure shape of the device channel and gate electrode [7–12] and the gate dielectric materials with different work functions. The characteristic analysis and structure optimization of the gate dielectric material [13–15] and gate dielectrics with different dielectric constants have been performed [15–20]. In device physics, the analytical modeling of TFETs with the double-gate structure [21–27] and surrounding-gate structure [28–33] has also been extensively performed. One disadvantage of silicon-based TFETs compared to MOSFETs is the smaller forward current, and the magnitude of the forward current is determined by the efficiency of the tunneling current generation. The tunneling current generation efficiency can be increased by reducing the band gap between valence band and conduction band in the region that is used to generate the band-to-band tunneling current or by reducing the thickness of the tunneling region. Therefore, in material engineering, TFET devices based on narrow-band gap semiconductor materials and heterojunction tunneling structures have been extensively developed [34–38]. Meanwhile, the introduction of two-dimensional materials into TFETs as tunneling layers with ultrathin thickness has been extensively studied [39–44]. In addition, some papers have reported the reliability of TFETs, such as the effect of source doping on tunneling band gap interleaving [45], the effect of trap-assisted tunneling on the subthreshold characteristics of TFETs [46], and the effect of random doping on the device performance perturbation [47]. However, the current research results mainly aim at the basic working characteristics and working principles of single TFETs, and the most important fundamental purpose of the research and development of TFETs is to provide a basic structural unit with lower power consumption and replace the existing MOSFET structure. To achieve this fundamental goal, it must be set in a specific circuit to verify its compatibility with MOSFET technology. At present, research on the circuit design strategy based on TFET devices is gradually conducted, such as the analog and mixed signal circuit [48–50], digital logic circuit [50, 51], power management circuit design [52]. There are also studies on the design of hybrid circuits based on MOSFETs and TFETs [53]. However, the doping types of the source region and drain region are opposite to each other, which creates an asymmetry of source region and drain region. This asymmetric structure makes it impossible to completely replace MOSFET with the source/drain symmetry.

Take the *n*-type TFET as an example. The side with *p*-type impurity is used as the source region, while the other side with *n*-type impurity is used as the drain region. When the device works, a positive potential difference must be applied from the drain region to the source region. If the source electrode and drain electrode are interchanged, i.e., the *p*-type impurity region is set at a higher potential relative to the *n*-type impurity region, then the PN junction formed by the *p*-type impurity region and *n*-type impurity region will always be in the positive bias state, which causes the failure of the control function of the gate electrode, the TFET will be almost always in the on state and cannot be turned off. In other words, it causes the failure of the TFET switch function. In other words, the circuit functional modules (such as transmission gates), which must use the bidirectional switching characteristics of transistors to work normally, are difficult to realize using conventional TFETs with an asymmetrical structure of source and drain, in order to solve these problems, we proposed a source drain symmetric and interchangeable bidirectional TFET (B-TFET) [54], which shows the advantage of bidirectional switching characteristics and compatibility with CMOS integrated circuits compared to the traditional asymmetrical TFETs. In this paper, we proposed a modified bilateral gate-controlled B-TFET with a planar channel. The effects of key structural parameters, such as the doping concentrations of the N^+^ region and P^+^ region, length of the N^+^ region and length of the intrinsic region, on the device performances, e.g., the transfer characteristics, *I*_on_–*I*_off_ ratio and subthreshold swing, are explained in detail based on physical analysis. Thereafter, these key structural parameters are optimized.

## Methods

**Fig. 1:**
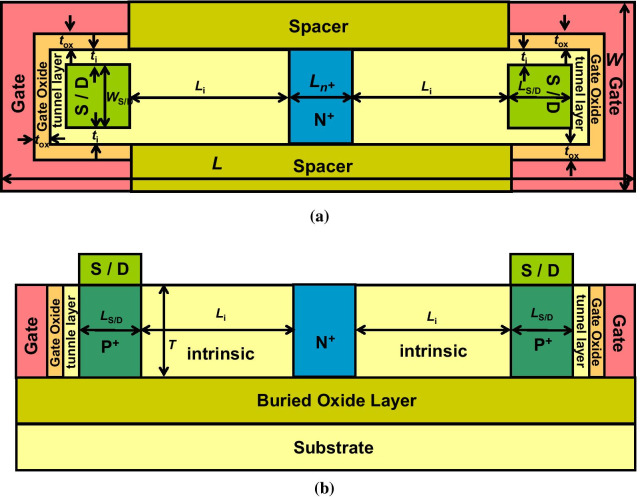
**a** Schematic top view of the bilateral gate-controlled N-Type B-TFET with planar channel. **b** Cross-view of the bilateral gate-controlled N-Type B-TFET

Figure 1a shows a schematic top view of the bilateral gate-controlled N-Type B-TFET with a planar channel. Figure 1b shows a cross-view of the bilateral gate-controlled N-Type B-TFET. Unlike the conventional TFET, the proposed B-TFET is completely symmetric, the source/drain interchangeable P^+^-doped regions lay on each side of the silicon body, and the gate electrode lays on both sides of the silicon body. The entire device structure is symmetric. The N^+^-doped region is in the central part of the silicon body. *L* and *W* are the entire length and entire width of the proposed device, respectively. *L*_i_ is the length of the intrinsic region; *L*_N+_ is the length of the N^+^ region; *L*_S/D_ and *W*_S/D_ are the length and width of the P^+^ source/drain interchangeable regions, respectively; $T$ is the silicon body thickness; $t_{{{\text{ox}}}}$ is the thickness of the gate oxide; $t_{i}$ is the thickness of the intrinsic tunnel region between S/D region and gate oxide.

In this paper, all physical models such as the Fermi statistic model, CVT mobility model, Auger recombination model, band-gap-narrowing model and a standard band-to-band tunneling model are turned on. All parameters of the device in this paper are listed in Table 1.

**Table 1:** Adopted device parameters

Parameters	Values
Body thickness (*T*)	100 nm
Gate oxide thickness ($t_{{{\text{ox}}}}$)	1 nm
The thickness of the tunnel region ($t_{i}$)	0.5 nm
The entire width of the proposed B-TFET (*W*)	13 nm
The length of the S/D interchangeable regions (*L*_S/D_)	8 nm
S/D region width (*W*_S/D_)	8 nm
N +-doped region length (*L*_N+_)	From 2 to 160 nm
The length of the intrinsic region between N^+^-doped region and P^+^-doped region ($L_{i}$)	From 4 to 100 nm
The thickness of the buried oxide layer	50 nm
Doping concentration of P^+^ region ($N_{A}$)	From $5 \times 10^{18}$ to $1 \times 10^{21} \,{\text{cm}}^{ - 3}$
Doping concentration of N^+^ region ($N_{D}$)	From $5 \times 10^{18}$ to $1 \times 10^{21} \,{\text{cm}}^{ - 3}$
Drain to source voltage ($V_{{{\text{ds}}}}$)	0.5 V
Gate to source voltage ($V_{{{\text{gs}}}}$)	From − 0.4 to 1 V

## Results and Discussion

**Fig. 2:**
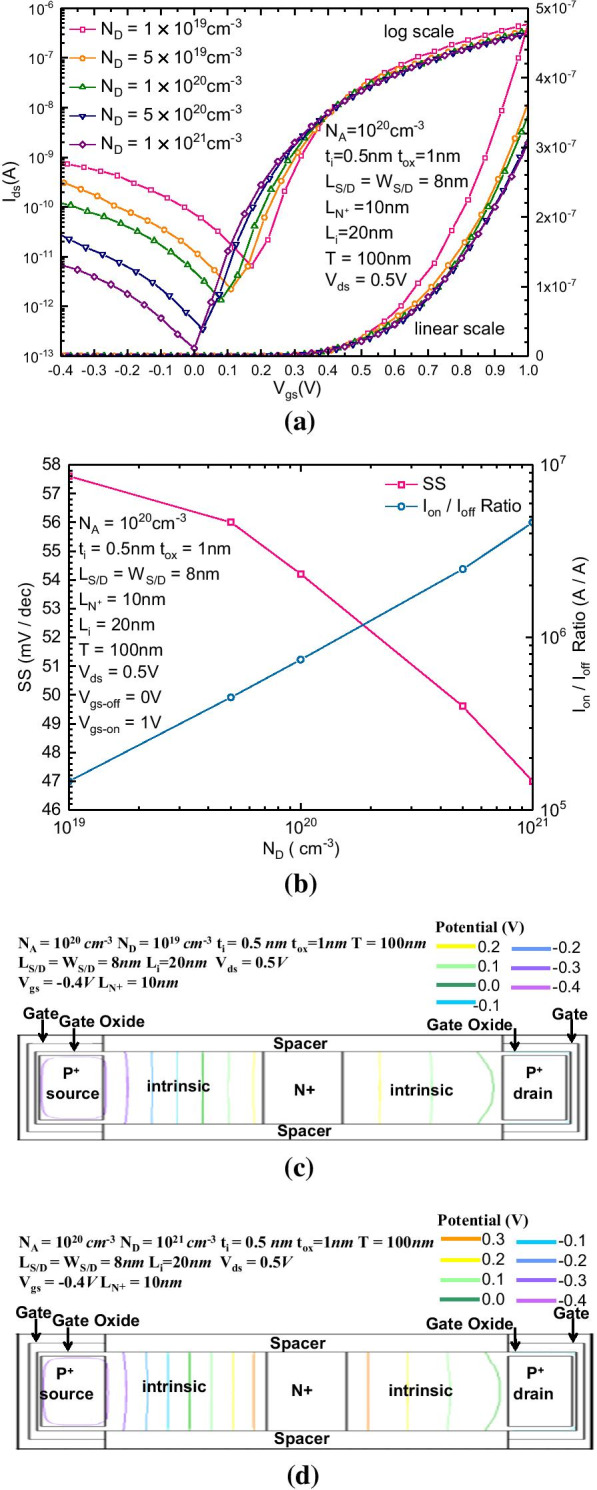
**a** $I_{{{\text{ds}}}}{-}V_{{{\text{gs}}}}$ transfer characteristics and **b** variation in SS and the $I_{{{\text{on}}}}{-}I_{{{\text{off}}}}$ ratio of the proposed B-TFET with different $N_{D}$; the reversely biased 2-dimensional potential distribution with **c** *N*_*D*_ = 10^19^ cm^−3^ and **d** *N*_*D*_ = 10^21^ cm^−3^

Figure 2a, b show the transfer characteristic, $I_{{{\text{on}}}}{-}I_{{{\text{off}}}}$ ratio and average SS with different $N_{D}$ ($10^{18}$–$10^{21} \,{\text{cm}}^{ - 3}$). In Fig. 2a, $N_{D}$ affects the intensity of the reversely biased drain-to-source leakage current. With the increase in doping concentration, the leakage current is significantly suppressed, and the forward current does not significantly change. In Fig. 2b, the SS and $I_{{{\text{on}}}}{-}I_{{{\text{off}}}}$ are also affected by $N_{D}$. With the increase in doping concentration, because the reverse leakage current is significantly suppressed, the current at the static operating point decreases, so the average SS also decreases. Because the forward current is much less affected than the reverse leakage, the $I_{{{\text{on}}}}{-}I_{{{\text{off}}}}$ ratio increases with the increase in doping concentration. Figure 2c, d show the 2-dimensional potential distributions of the proposed B-TFET with $N_{D}$ equal to 10^19^ cm^−3^ and 10^21^ cm^−3^, respectively. When the gate electrode is reversely biased, a strong electric field will be generated between the forward biased drain electrode and the reverse biased gate electrode, which results in a strong band-to-band tunneling near the drain region. Among the resulting generated electron–hole pairs, the electrons can directly flow out of the drain electrode, while the valence band holes must flow through the N^+^ region, subsequently to the intrinsic region in the source side and be discharged by the source electrode to form the continuous leakage current. To minimize the leakage current, the holes produced by band-to-band tunneling should be effectively blocked from flowing out of the N + region. Compared with the N + region with lower concentration, the N + region with higher concentration forms a larger potential difference between P + region and N + region, i.e., the potential value at the boundary between the intrinsic region and the N + region will increase with the increase in $N_{D}$ because the N + region with higher concentration can produce a larger electronic concentration difference between source and drain. Then, more electrons can be diffused from the N + region to the intrinsic regions on both sides of the N + region, which increases the amount of positive charge (mainly composed of donor) in the N + region after ionization and consequently increases the potential difference between the P + region and N + region. Precisely because the N + region with higher doping concentration has a higher potential than both the source and drain sides after ionization, the holes generated by the band-to-band tunneling near the drain region can be more effectively blocked, which more effectively decreases the leakage current.

**Fig. 3:**
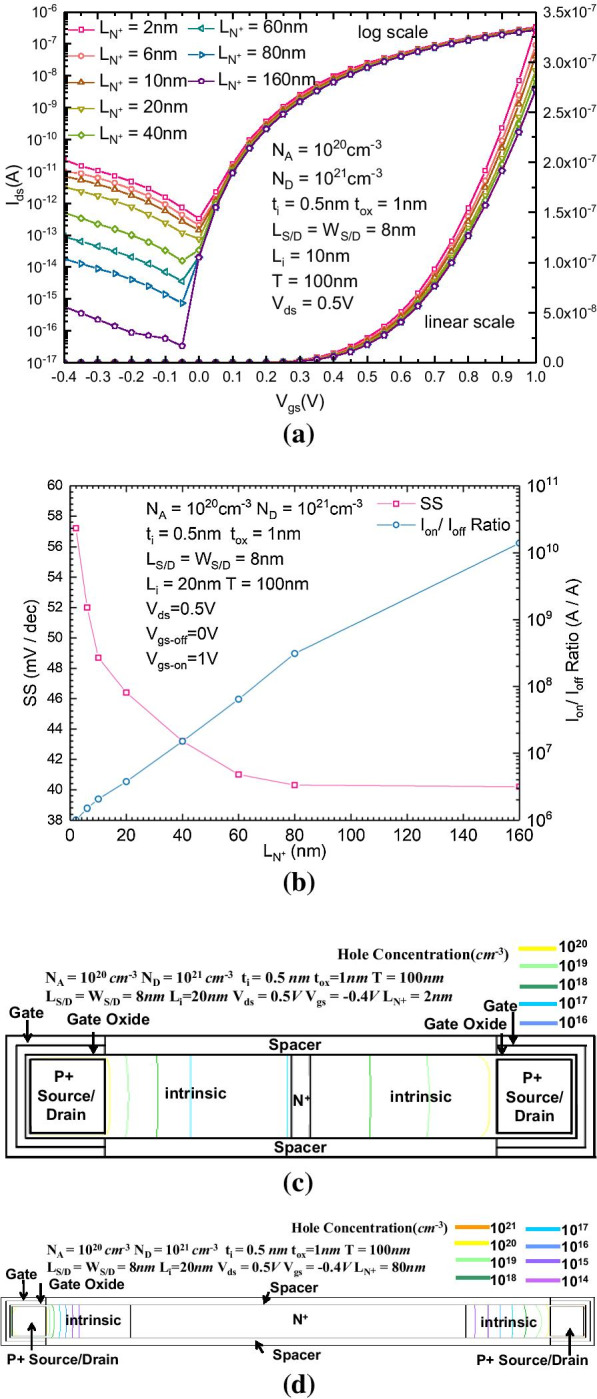
**a** $I_{{{\text{ds}}}}{-}V_{{{\text{gs}}}}$ characteristics; **b** variation in the SS and the $I_{{{\text{on}}}}{-}I_{{{\text{off}}}}$ ratio of the proposed B-TFET with different L_N+_; 2-dimensional hole concentration distribution of the proposed B-TFET under reversely biased for *L*_N+_ equal to (3) 2 nm and (4) 80 nm

In addition to the doping concentration of the N + region, another key parameter of the N + region, which can significantly affect the reversely biased leakage current, is the length of the N + region. Figure 3a, b show the $I_{{{\text{ds}}}}{-}V_{{{\text{gs}}}}$ transfer characteristics of the proposed B-TFET with different *L*_N+_. The reversely biased leakage current largely decreases with increasing *L*_N+_. As Fig. 2b shows, the subthreshold swing and $I_{{{\text{on}}}}{-}I_{{{\text{off}}}}$ are also affected by *L*_N+_. With the increase in *L*_N+_, because the reverse leakage current is significantly suppressed, the current at the static operating point and average SS are also reduced. The forward current is far less affected than the reverse leakage, and the $I_{{{\text{on}}}}{-}I_{{{\text{off}}}}$ ratio increases with the increase in *L*_N+_. Figure 3c, d show the 2-dimensional hole concentration distribution of the proposed B-TFET with *L*_N+_ equal to 2 nm and 80 nm, respectively. When *L*_N+_ is equal to 2 nm, the minimal hole concentration in the N + region is larger than 10^17^ cm^−3^, while when *L*_N+_ is equal to 80 nm, the minimal hole concentration is less than 10^14^ cm^−3^. The increase in length of the N + region enhances its ability to prevent holes from passing through the N + region. As a non-equilibrium minority carrier in the N + region, when the N + region is longer, more holes will be recombined with electrons before passing through the N + region, so the increase in length of the N + region can also form a continuous reversely biased leakage current. The average SS can be reduced to 40.2 mV/dec, and the $I_{{{\text{on}}}}{-}I_{{{\text{off}}}}$ ratio can exceed 10^10^.

**Fig. 4:**
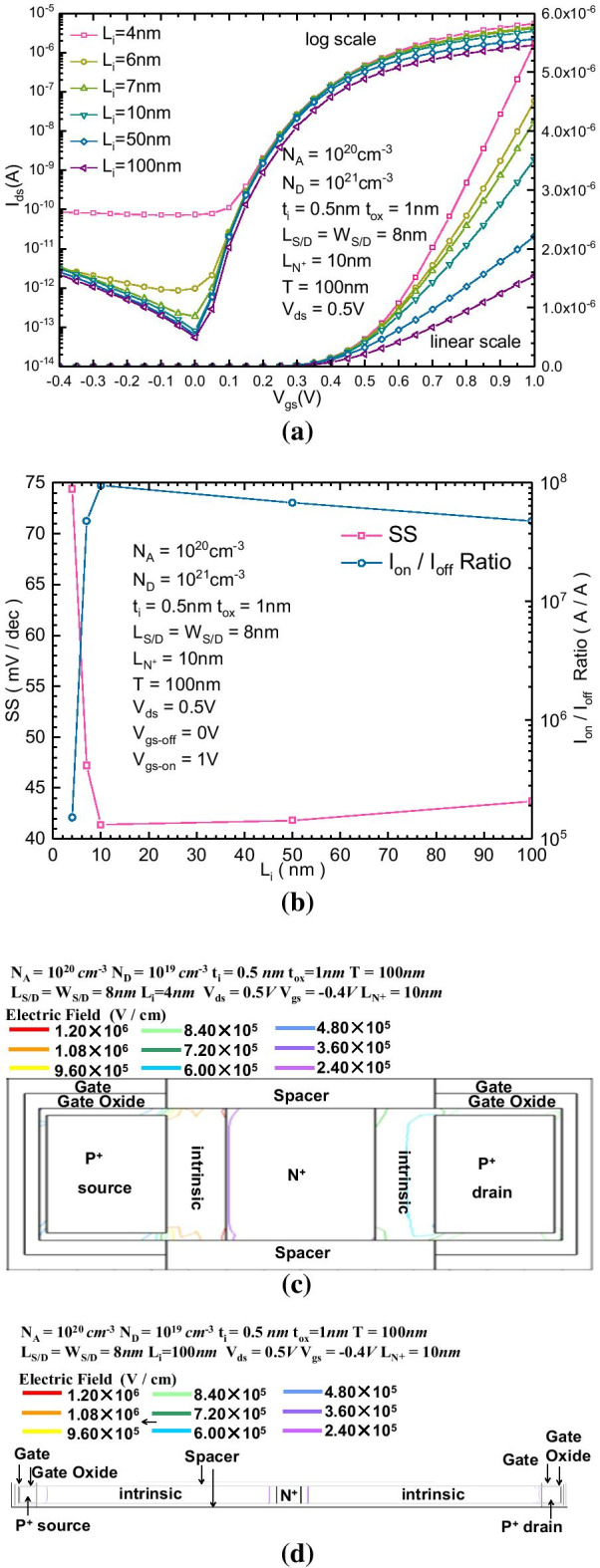
**a** $I_{{{\text{ds}}}}{-}V_{{{\text{gs}}}}$ characteristics of B-TFET and **b** variation in SS and the $I_{{{\text{on}}}}{-}I_{{{\text{off}}}}$ ratio with different $L_{i}$; 2-dimensional reversely biased potential distribution of the proposed B-TFET for *L*_*i*_ equal to **c** 20 nm and **d** 100 nm

Figure 4a, b show the $I_{{{\text{ds}}}}{-}V_{{{\text{gs}}}}$ transfer characteristics and changes in SS and $I_{{{\text{on}}}}{-}I_{{{\text{off}}}}$ ratio of the proposed B-TFET with different *L*_i_, respectively. The forward current decreases with increasing *L*_i_ because the resistance of the intrinsic region is proportional to the length of itself. Then, to maximize the forward current, the length of the intrinsic region should be minimized. However, the decrease in length of the intrinsic region enhances the electric field in the intrinsic region between the source P + region and the N + region, so the band bending near this region is larger than the intrinsic region near the drain electrode, which induces more reversely biased leakage current. Figure 4c, d show the 2-dimensional reversely biased potential distribution of the proposed B-TFET for *L*_i_ equal to 4 nm and 100 nm, respectively. For the shortest *L*_i_ (4 nm) case, the electric field in the intrinsic region between the source P + region and the N + region near the source electrode is much stronger than that in the intrinsic region between the drain P + region and the N + region near the drain electrode. Then, the leakage current almost remains constant, which is independent of the change in gate voltage. Figure 4b shows that the optimal value range of *L*_i_ is approximately 7–10 nm, where the SS decreases to a valley value of 41 mV/dec and the $I_{{{\text{on}}}}{-}I_{{{\text{off}}}}$ ratio increases to a maximum value of almost 10^8^.

**Fig. 5:**
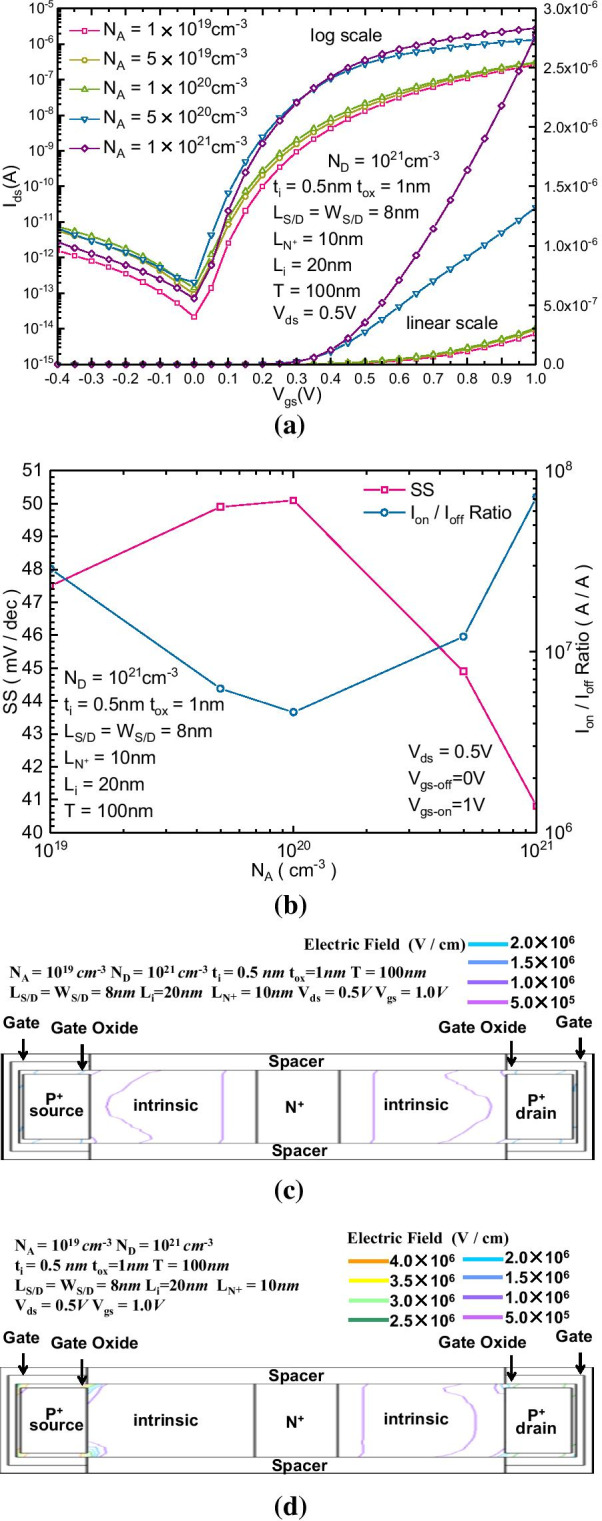
**a** $I_{{{\text{ds}}}}{-}V_{{{\text{gs}}}}$ transfer characteristics, **b** variation in SS and $I_{{{\text{on}}}}{-}I_{{{\text{off}}}}$ ratio of the proposed B-TFET with different $N_{A}$. Two-dimensional reversely biased electric field distribution of the proposed B-TFET for $N_{A}$ equal to (3) 10^19^ cm^−3^ and (4) 10^21^ cm^−3^

Figure 5a, b show the $I_{{{\text{ds}}}}{-}V_{{{\text{ds}}}}$ transfer characteristics and change in SS and $I_{{{\text{on}}}}{-}I_{{{\text{off}}}}$ ratio of the proposed B-TFET with different $N_{A}$. Figure 5a shows that by increasing the concentration of the P + -doped region, we can obtain less SS and a larger forward current. The reversely biased leakage current is not obviously affected by the change in $N_{A}$, but the forward current can be increased with the increase in $N_{A}$. In Fig. 5b, both SS and $I_{{{\text{on}}}}{-}I_{{{\text{off}}}}$ ratio can be improved by increasing $N_{A}$. Figure 5c, d show the 2-dimensional electric field distribution of the proposed B-TFET with $N_{A}$ equal to 10^19^ cm^−3^ and 10^21^ cm^−3^, respectively. The increase in $N_{A}$ enhances the electric field in the intrinsic tunnel region; then, more electron- hole pairs can be generated through band-to-band tunneling, which enhances the forward current of the proposed B-TFET.

**Fig. 6:**
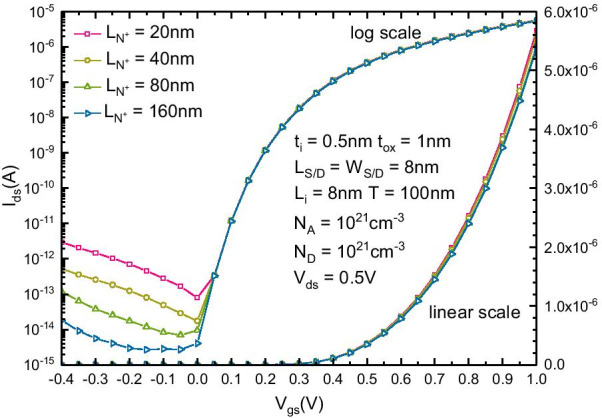
$I_{{{\text{ds}}}}{-}V_{{{\text{ds}}}}$ transfer characteristics of the optimized B-TFET with different L_N+_

According to the above discussion, both $N_{D}$ and $N_{A}$ should be set to the maximal possible value. The optimal value range of *L*_i_ is 7–10 nm. However, there is a tradeoff between the static power consumption and *L*_N+_. Figure 6 shows the $I_{{{\text{ds}}}}{-}V_{{{\text{ds}}}}$ transfer characteristics of the optimized B-TFET with different *L*_N+_. *L*_N+_ can be selected according to different static power consumption design requirements. As a compromise, to ensure that the $I_{{{\text{on}}}}{-}I_{{{\text{off}}}}$ ratio is above 10^8^, L_N+_ is recommended to be above 20 nm. The on current is increased to approximately 6 × 10^–6^ A, and the SS is reduced to 38 mV/dec.

## Conclusions

In this paper, the effects of the structural parameters and internal mechanism of a bilateral gate-controlled S/D symmetric and interchangeable bidirectional tunneling field effect transistor are analyzed. The effects of the key parameters such as the concentration and length of the N + region, length of the intrinsic region between the P + and N + regions, and concentration of the P + region have been discussed in detail. Compared with the conventional TFET, the B-TFET has the advantage of strong resistance to the reversely biased leakage current. Thereafter, good performance such as a lower average SS and a higher $I_{{{\text{on}}}}{-}I_{{{\text{off}}}}$ ratio can be obtained. Moreover, due to the structural symmetry and source/drain interchangeable and bidirectional switching characteristics, it is more compatible with the CMOS circuit.

## Data Availability

We included a statement of availability of data and material for ourselves and on behalf of our coauthors under the “Competing interests”. All available data and material are original work. All data have been clearly provided in the manuscript without additional data and supporting materials.

## Abbreviations

*L*: Entire length of the proposed device
*W*: Entire width of the proposed device
*L*_i_: Length of the intrinsic region
*L*_N__+_: Length of the N^+^ region
*L*_S__/__D_: Length of the P^+^ source/drain interchangeable regions
*W*_S__/__D_: Width of the P^+^ source/drain interchangeable regions
$T$: Silicon body thickness
$t_{{{\text{ox}}}}$: Thickness of the gate oxide
$t_{i}$: Thickness of the intrinsic tunnel region between S/D region and gate oxide
MOSFET: Metal oxide semiconductor field effect transistor
TFET: Tunnel field effect transistor
